# More severe disturbance regimes drive the shift of a kelp forest to a sea urchin barren in south-eastern Australia

**DOI:** 10.1038/s41598-020-67962-y

**Published:** 2020-07-09

**Authors:** Paul E. Carnell, Michael J. Keough

**Affiliations:** 1 School of BioSciences, The University of Melbourne, Parkville, VIC 3010 Australia; 2 Centre for Integrative Ecology, School of Life and Environmental Sciences, Deakin University, Geelong, VIC 3216 Australia

**Keywords:** Ecosystem ecology, Population dynamics, Community ecology

## Abstract

Climate change is influencing the frequency and severity of extreme events. This means that systems are experiencing novel or altered disturbance regimes, making it difficult to predict and manage for this impact on ecosystems. While there is established theory regarding how the frequency of disturbance influences ecosystems, how this interacts with severity of disturbance is difficult to tease apart, as these two are inherently linked. Here we investigated a subtidal kelp (*Ecklonia radiata*) dominated community in southern Australia to assess how different disturbance regimes might drive changes to a different ecosystem state: sea urchin barrens. Specifically, we compared how the frequency of disturbance (single or triple disturbance events over a three month period) influenced recruitment and community dynamics, when the net severity of disturbance was the same (single disturbance compared to triple disturbances each one-third as severe). We crossed this design with two different net severities of disturbance (50% or 100%, kelp canopy removal). The frequency of disturbance effect depended on the severity of disturbance. When 50% of the canopy was removed, the highest kelp recruitment and recovery of the benthic community occurred with the triple disturbance events. When disturbance was a single event or the most severe (100% removal), kelp recruitment was low and the kelp canopy failed to recover over 18 months. The latter case led to shifts in the community composition from a kelp bed to a sea-urchin barren. This suggests that if ecosystems experience novel or more severe disturbance scenarios, this can lead to a decline in ecosystem condition or collapse.

## Introduction

Ecosystems are under increasing pressure from both direct and indirect human influences^1,2^. Examples include land clearing for agriculture, altered fire regimes, the intensified usage of marine and coastal areas and a range of impacts from climate change^3,4^. Climate change is predicted to change average annual environmental conditions such as temperature and rainfall, but also influence extreme events such as storms, cyclones, droughts and floods. It is estimated that 20% of extreme rain events and 75% of all hot temperature extremes can be attributed to climate change^5^. Such conditions mean that ecosystems are experiencing novel or altered disturbance regimes, providing new challenges for those charged with managing biodiversity and ecosystem services. Responding to these challenges demands that we adapt our understanding of natural systems to consider how a change in the severity or frequency of disturbance events might influence species and ecosystem dynamics^6-8^.

Natural disturbance plays a crucial role in maintaining species diversity and ecosystem function in ecosystems^9-12^. Disturbances cause mortality, free up resources and generate local changes in community structure^13-15^. While the Intermediate Disturbance Hypothesis predicts ecosystems to have relatively low species diversity at either ‘low’ or ‘high’ disturbance regimes^10,16^, less than 20% of studies since then have found support for it^17-19^. In part, uncertainties in this disturbance-diversity relationship arise because a disturbance regime can be split into different components. This includes the damage caused by the severity of the disturbing force (e.g. 50% or 100% of a given area), and frequency (number of disturbances per unit time)^11^.

The severity of a disturbance strongly influences species recolonisation and community development of disturbed areas^20-23^. The response of the community can then depend on the life histories of component species, and the influence of early colonisers on the community^8,24^. While some species may recover better at moderate levels of disturbance, others may require a severe event to trigger reproduction or to be able to maintain space long enough to reproduce^25-27^. However, these responses also depend heavily on the frequency of disturbance^28^. For example, the resilience^29^, diversity^30^ and food-web structure^7^ of systems often become eroded with multiple (frequent) disturbances compared to a one off (infrequent) disturbance^21,31,32^. Recently, attempts have been made to combine multiple disturbance theories into a coherent framework that allows for multiple disturbance pathways and interactions^8^.

In natural ecosystems, frequency and severity of disturbance often co-vary^33,34^. For example, more severe disturbances tend to occur less frequently^35-37^. However, more frequent less severe disturbances may have the same net severity of disturbance (three disturbances of 33% of plant biomass) as one more severe disturbance event (one disturbance of 100% of plant biomass). While the net biomass loss might be the same, this difference in how a patch reached its current state could have important ramifications on the recovery or stability of an ecosystem^38^. Here, we define this combination of frequency and severity of disturbances as the disturbance regime; where three low severity disturbances (triple-low) are equal to one high severity disturbance (single-high).

In kelp forests, the disturbance regime is driven by spatial or temporal variation in storm frequency and severity. Here, differences in the disturbance regime can often override top-down or bottom-up effects on primary productivity^39^, and shifts in the disturbance regime can lead to more simplified food webs^7^. In temperate Australia, the kelp *Ecklonia radiata* (hereafter *Ecklonia*) forms dense monospecific or mixed-species canopy beds, generally < 1 m above the sea bed, which support a diverse community of algae, invertebrates and fishes^40^. Here, the disturbance regime can also drive a change in this ecosystem from productive kelp forest to sea urchin barren, without an increase in sea urchin numbers^41^. This may occur as the ecological processes responsible for initiation of a community shift may be quite different from the processes needed to maintain that state or to reverse it^42-44^.

In order to disentangle the interdependent nature of the frequency and severity of disturbance on ecosystem stability and resilience, we aimed to understand how a single severe disturbance event may differentially influence kelp recovery and community dynamics compared to three low severity disturbances. We focused on the subtidal kelp (*Ecklonia radiata*) dominated community, where the frequency and intensity of storm events varies, often creating small canopy gaps and occasionally damaging large areas^45^. In this kelp community, we assumed the three low severity disturbances to more closely approximate the natural disturbance regime. In this experiment we compared *Ecklonia* recruitment and benthic species and community dynamics in response to: (a) the impact of a single high severity disturbance, compared to three low severity disturbances, resulting in the same net severity of disturbance, (b) different net-severities of disturbance (50% and 100%). Our design enabled testing for both main effects of each of these effects and for interactions between these two. Here, we expected recruitment and recovery of *Ecklonia* to be lower in the more severe disturbance regime (a single high severity disturbance event) compared to three low severity disturbances. A prior disturbance experiment at a nearby location suggested that *Ecklonia* may not recover from a single 100% disturbance event, but would maintain its level of cover at 50% disturbance^41^. We also expected the community dynamics to follow similar patterns dependent on *Ecklonia*’s response.

## Materials and methods

### Site information

The experiment was conducted in kelp beds at Mt. Eliza, Victoria, Australia (38° 10′30.10″ S, 145° 4′30.01″ E). There the bottom is 3–4 m deep, covered predominantly by the common kelp *Ecklonia*, which dominates many temperate subtidal reefs in Australia and New Zealand. Like other kelp species, it provides habitat for a range of species by influencing light, sedimentation and water movement^46-49^. The average density (± SEM) of *Ecklonia* holdfasts on the reef prior to disturbance was 9.3 ± 0.2 m^−2^, with an average canopy cover of 70.8 ± 1.4%.

The reef also includes a mosaic of canopy-forming brown algal species from the order Fucales (fucoids), including *Caulocystis uvifera* and several *Sargassum* and *Cystophora* species, which generally reside in the gaps in the *Ecklonia* canopy. These gaps are thought to be formed by storms that dislodge kelps. The detached plants entangle with other kelps, leading to further dislodgment^45^. In the clearings formed from these events, the substratum is also covered by turf-forming ectocarpales, dictyotalean algae, filamentous red algae, encrusting coralline algae, sessile invertebrates and sediment. Although storm events likely remove a range of foliose macroalgae, we focused the disturbance treatments on the removal of the dominant kelp *Ecklonia* and how this influenced the algal and sessile invertebrate community.

### Experimental manipulations

**Figure 1:**
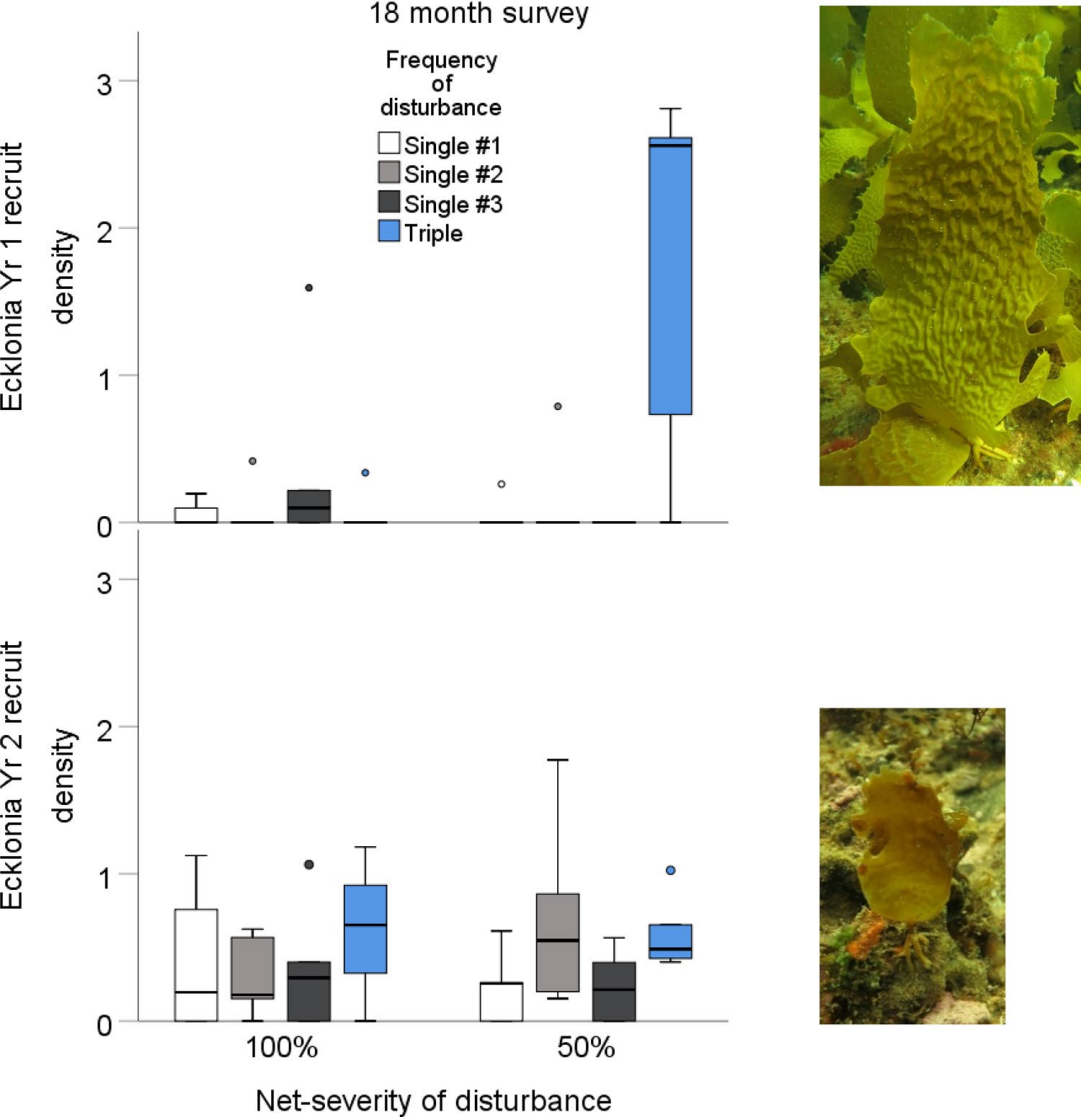
The mean density per m^2^ in plots of the kelp *Ecklonia radiata* m^−2^ recruits from Year 1 or Year 2 at 18 months post-disturbance by the net-severity of disturbance. The different colours represent the different frequency of disturbance treatments, single disturbance in the first month(#1) = white, single disturbance in the second month (#2) = grey, single disturbance in the third month (#3) = dark grey and the triple disturbances of a third of the net-severity = blue.

To tease apart the impacts of disturbance severity and frequency experimentally, over a 3 month period one high severity or three low severity disturbance events resulting in the same net disturbance were created (Fig. 1). This design was crossed with two different disturbance severities, a total of 50% or 100% of *Ecklonia* removed. For the frequency treatments, the comparison of interest was between one high-severity disturbance (hereafter termed single) and three low-severity disturbances (hereafter termed triple). We included temporal controls to account for any potential differential response resulting simply from the single disturbance occurring at different points in time (Table 1). We did this by conducting separate high-disturbances at the same time as each of the three low-disturbances. For ease of reference, the single high-disturbance treatments conducted at the different time points are hereafter termed “once #1” for the first disturbance time point, “once #2” for the second, and “once #3” for the third (Table 1). This resulted in a total of eight treatments.

**Table 1:** Experimental design testing how the frequency of disturbance (one or three disturbance events) over a three month period influenced recruitment and community dynamics, when the net severity of disturbance was the same (one disturbance of 100% compared to three disturbances of 33%).

Frequency of disturbance	Timing control	Net-Severity	Month #1	Month #2	Month #3
Triple		50%	17%	17%	17%
		100%	33%	33%	33%
Single	#1	50%	50%		
		100%	100%		
	#2	50%		50%	
		100%		100%	
	#3	50%			50%
		100%			100%

Additionally, this was crossed with two different net severities of disturbance (50% or 100%, kelp canopy removal). To account for potential differences due to the timing of disturbance, we implemented timing controls whereby in each month (#1, #2 or #3) a new treatment plot was disturbed once. N = 40, 5 replicates for each treatment. Each row of this table represents one of the eight treatments deployed in the experiment.

Five replicates were established per treatment combination (total n = 40), and were distributed randomly on the available reef, separated by at least five metres. Plots were roughly 2 × 2 m, but with variation driven by reef topography, the mean (and 1 SEM) plot size was 5.0 ± 0.1 m^2^. Plots were set up and initial surveys conducted in the austral summer of 2011/12. Experimental treatments were applied through February–April 2012, just before peak *Ecklonia* sporophyte recruitment in winter (June–August:^45,50^). Kelp was removed by cutting the stipe just above the holdfast, a practice shown to have no additional artefacts compared to removing the entire kelp including the holdfast^51^. The experimental removals occurred just prior to the period of more intense storm disturbance to kelp beds in south-eastern Australia, which tends to occur in winter and spring^45^.

### Survey methods

Responses to the experimental treatments were measured using four randomly placed 0.25 m^2^ quadrats in each plot. Each quadrat was photographed and percentage cover of *Ecklonia* and other algal and invertebrate groups was quantified using the image analysis program CPCe^52^. As the % cover was just done from photos, species falling underneath a canopy are not taken into account. A point-intercept method was used to estimate percentage cover by identifying the species under fifty randomly allocated points per quadrat. Other than *Ecklonia*, species were grouped into broader morphological (following^53^) or taxonomic groupings during surveys. This included canopy-forming fucoids (*Caulocystis uvifera*, *Sargassum* spp. and *Cystophora* spp.), turf-forming Ectocarpales, Dictyotales, filamentous red algae, encrusting coralline algae, and sessile invertebrates (the coral *Plesiastrea versipora*, sponges, colonial ascidians, soft corals). The percentage of sediment covering the plots was also included.

Surveys of the benthic community were conducted monthly for the first 12 months. , but given the difference in the timing of treatments, we chose to conduct analyses at time points relative to the final disturbance of that treatment, rather than on each survey date . This means, when we compare temporal control treatments at 6 months post-disturbance, this is actually comparing surveys at different points in time, but the same number of months after their final disturbance. We then conducted a final survey roughly 18 months post-treatment, in November 2013, when all treatments were assessed at the same time.

In addition to the photo quadrats, *Ecklonia* recruits (< 12 months old), juveniles (12–24 months old) and adults (> 24 months old) were counted in each plot at 6, 12 and 18 months post-disturbance. We refer to *Ecklonia* recruits settled in the first recruitment season post-disturbance (winter 2012) as “year 1 recruits” while those settled in the second recruitment season post disturbance (winter 2013) are termed “year 2 recruits”.

### Analysis

We tested for differences in recruitment of *Ecklonia* using a two factor repeated measures ANOVA at 6, 12 and 18 months post-disturbance for “year 1 recruits” and tested differences for “year 2 recruits” using a 2-factor ANOVA at 18 months post-disturbance. We tested for differences in species abundance over time between treatments using two-factor repeated measures ANOVA for each of the three analysis scenarios with ‘severity’ and either ‘frequency’ or ‘timing’ of disturbance as fixed factors. Data from 0, 2, 4, 6, 12 months post-disturbance and the final survey in November 2013 (18 months) were included in this analysis. Data were logit-transformed to improve normality to meet the necessary statistical assumptions. The assumption of sphericity was tested using Mauchly’s test. When this assumption was violated, we used Greenhouse–Geisser adjusted *P* values (Table A1). All ANOVAs were conducted using SPSS V25.

We tested the influence of frequency and severity on overall algal assemblage structure (percentage cover of all taxonomic groups over time) using the PERMANOVA add-on for PRIMER 6^54^. Differences between treatments were analyzed by partly nested PERMANOVA for each experiment using unrestricted permutations of data with 9,999 permutations. These analyses used experimental treatments, with plots nested within treatments, and time as the within-plot factor. Principal coordinates ordinations (PCO) based on the Bray–Curtis dissimilarity matrix of percent covers of taxonomic groups were also used to visualize differences in overall community structure through time^54^.

## Results

### Recruitment

In Year 1, recruitment of *Ecklonia* in experimental plots varied with both frequency and severity of disturbance (Fig. 1, Table 2). Plots that experienced three disturbances of 17% had the most recruits over the period of the experiment, compared to when disturbances were more severe, either one 50% disturbance or 100% net-disturbance (Fig. 1). However, for Year 2 recruits neither frequency or severity of disturbance had an effect (Table 2, Fig. 1).

**Table 2:** Two-way repeated measures analysis of variance (ANOVA) of the severity and frequency of disturbance on the natural log transformed number of *Ecklonia* recruits that recruited in the first year (Yr 1 recruits) and the second year (Yr 2 recruits) at 6, 12 and 18 months post disturbance. *p* values < 0.05 are in bold.

Source	df	Yr 1 recruits 6, 12, 18 months			Yr 2 recruits 18 months		
		MS	F-ratio	*p*	MS	F-ratio	*p*
Severity (S)	1	1.287	5.450	**0.026**	0.006	0.081	0.778
Frequency (F)	3	0.541	2.293	0.097	0.117	1.676	0.192
S*F	3	1.673	7.084	**0.001**	0.057	0.821	0.492
Error	32	0.236			0.070		
Time (T)	2	23.68	117.9	** < 0.001**			
T*S	2	0.077	0.384	0.571			
T*F	2	0.583	2.907	**0.040**			
T*S*F	2	0.019	0.093	0.976			
Error	32	0.201					

### Percentage cover

**Figure 2:**
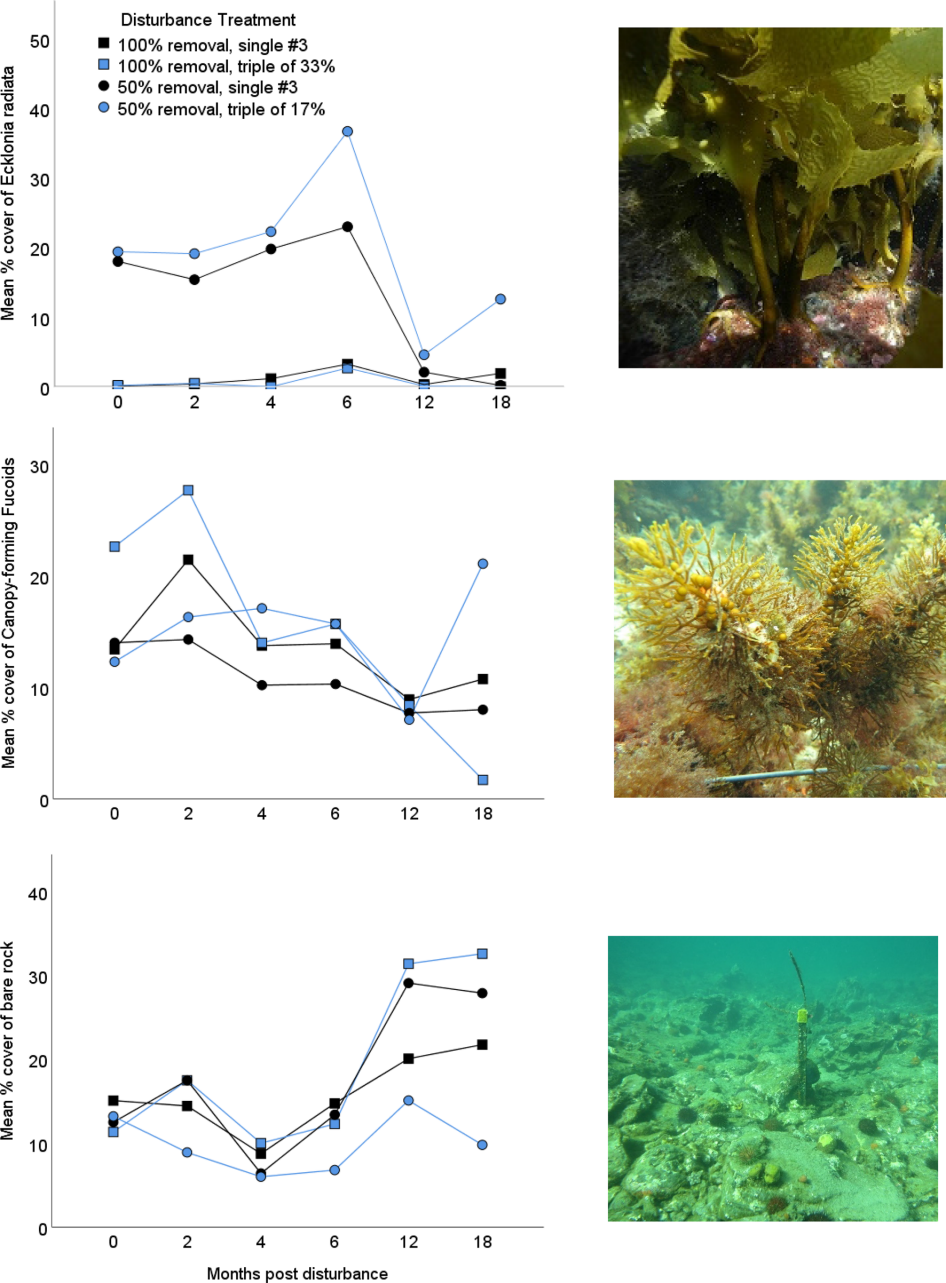
Average percentage cover over time (months post-disturbance) of *Ecklonia radiata*, canopy-forming fucoids and bare rock and the interaction between severity (50% or 100%) and frequency (single or triple) of disturbance. Here, just the single disturbance in the third month (#3) is compared to the triple disturbances of a third of the net-severity for ease of visualisation. Figures showing the once removal in the first and second month are shown in Figs. A1 and A2.

**Figure 3:**
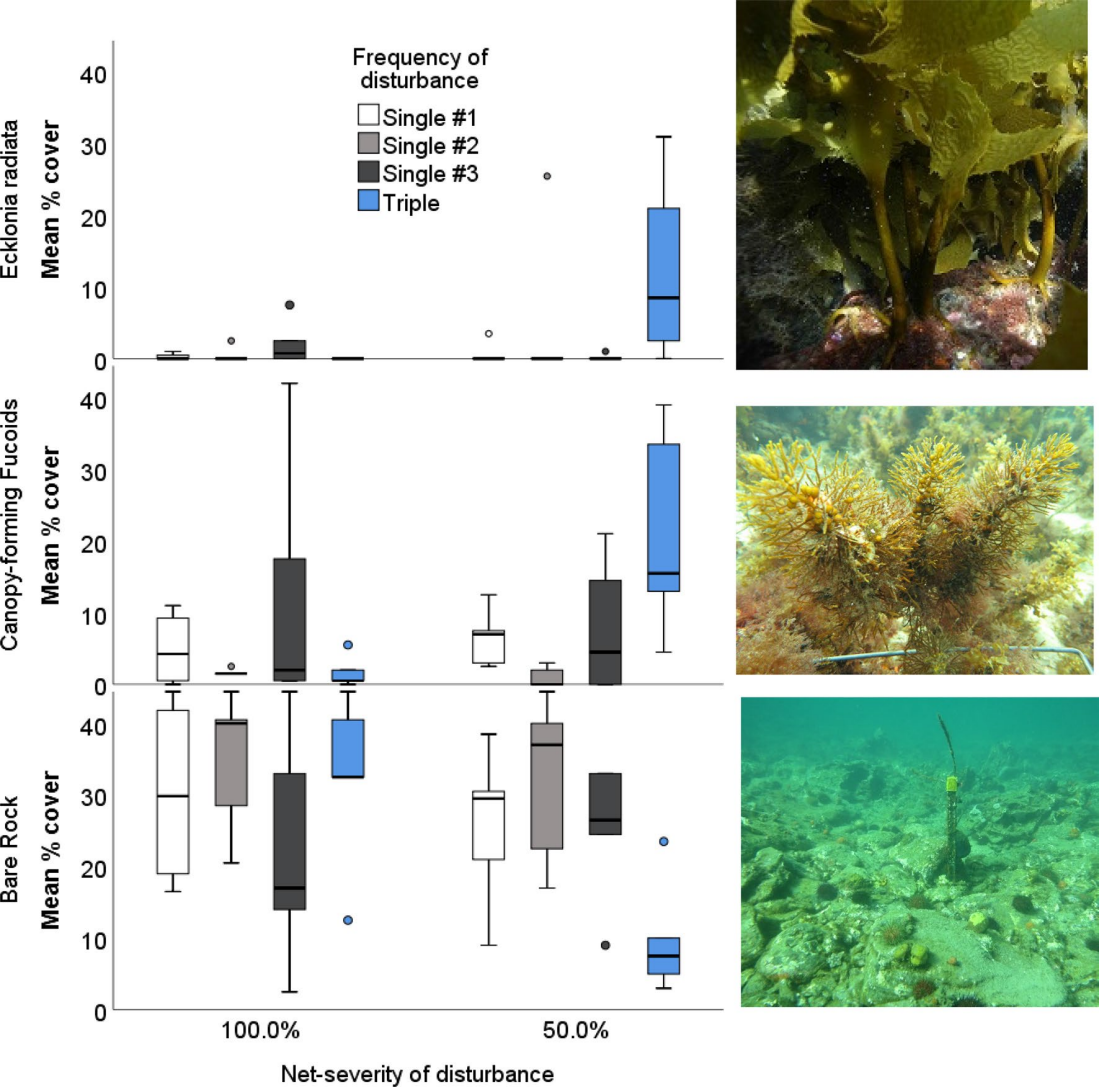
Impacts of the frequency and severity of disturbance on % cover of. *Ecklonia radiata,* canopy-forming fucoids and bare rock at the end of the experiment (18 month survey). The frequency of disturbance (single or triple disturbance events) over a three month period, where the net severity of disturbance was the same (single disturbance of 100% compared to triple disturbance of a third of the net-severity) Additionally, this was crossed with two different net-severities of disturbance (50% or 100%, kelp canopy removal). To account for potential differences due to the timing of disturbance, we implemented timing controls whereby in each month (single #1, single #2 or single #3) a new treatment plot was disturbed once.

Percent cover of *Ecklonia* was similarly influenced by the interaction between frequency and severity of disturbance, and varied over time (Fig. 2, Table 3). With 50% removal over three disturbance events, after 18 months *Ecklonia* was more abundant than in any other disturbance treatment (Fig. 3). Where all *Ecklonia* was removed, there was little increase in cover by the end of the experiment, regardless of disturbance frequency.

**Table 3:** Two-way repeated measures analysis of variance (ANOVA) of the severity and frequency of disturbance on the logit transformed percentage cover of species groups over the 18 month experimental period.

Species	Time (T)* frequency (F)	T* severity (S)	T*F*S	F*S	F*S 0, 2, 4, 6, 12, 18
*Ecklonia radiata*	**0.001**	** < 0.001**	**0.005**	0.129	
Canopy-forming Fucoids	0.007	**0.015**	**0.007**	0.396	
Bare rock	** < 0.001**	0.596	**0.020**	0.138	

*T*  Time, *F* Frequency, *S* Severity. *p* values < 0.05 are in bold. Summary table of results of each two-way ANOVA for the cover of various species. Each dot represents the six time points analysed: Initial, 2, 4, 6, 12, 18 months post final disturbance. A clear dot represents probabilities > 0.05 and black dot represents *p* < 0.05. No analyses were run and table cells are left blank for species that did not show an interaction with time. The residual df was 75 with urchin density included as a co-variate and 80 without.

The effect of frequency of disturbance on abundance of canopy-forming fucoids and bare space cover varied with the severity of disturbance, and through time (Table 3, Fig. 2). For the fucoids, this interaction stemmed from a greater cover in the 100% triple-low disturbance treatment initially, that disappeared in subsequent surveys (Table 3, Fig. 3). By the 18 month survey, similar to *Ecklonia*, the triple-low 50% net-severity disturbance treatment had the greatest cover (Fig. 3).

For bare space, cover initially was similar between treatments but by the final 18 month survey was greater in the single disturbance treatments compared to the triple disturbance treatment, but only where the net severity of disturbance was 50% (Fig. 2, Fig. 3).

### Community response

**Figure 4:**
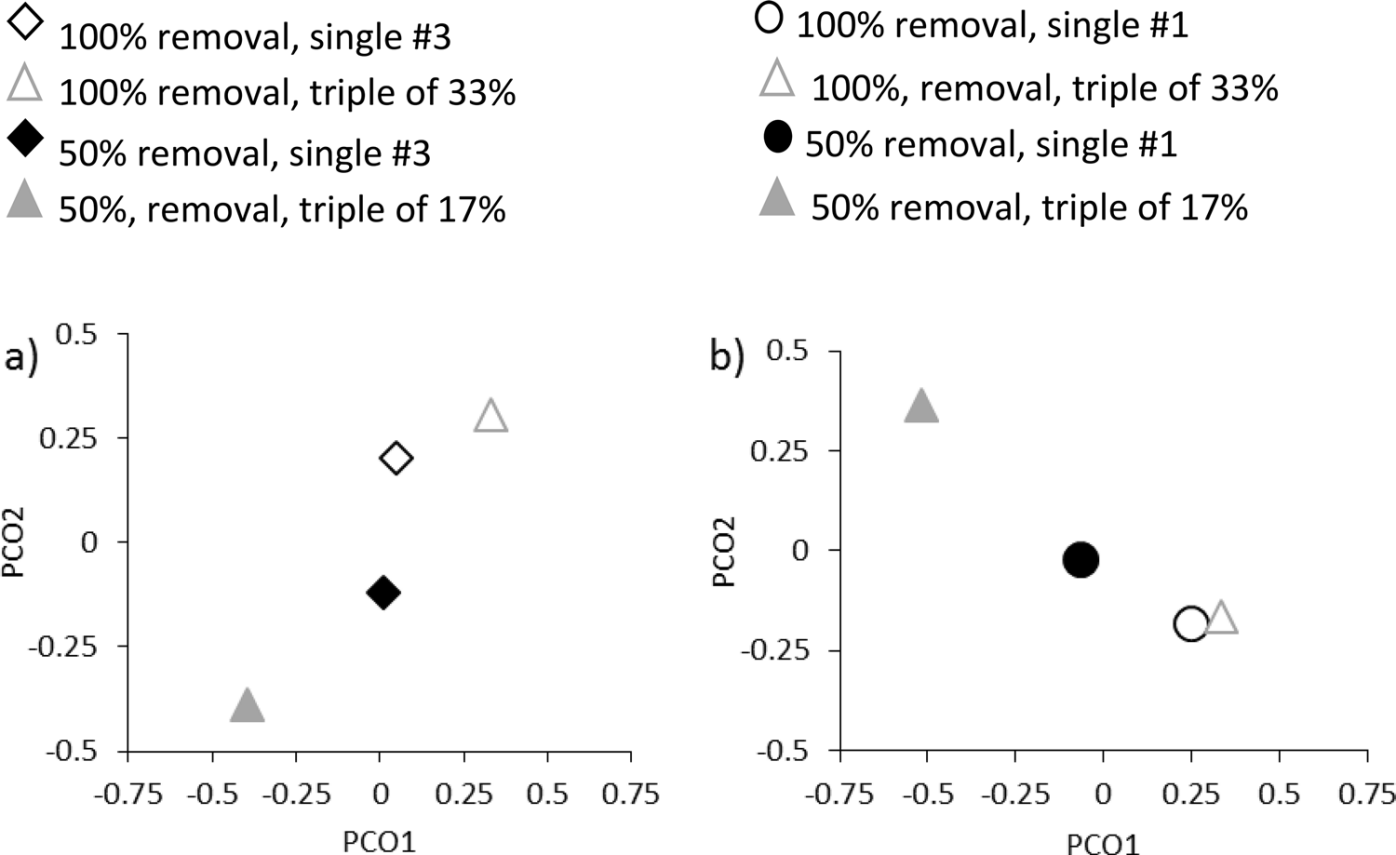
Principal coordinates ordination (PCO) of distances among centroids on the basis of the Bray–Curtis measures of percent cover to the frequency and severity of disturbance in a) single disturbance in month #3 and triple disturbances and b), single disturbance in month #1 and triple disturbances. Centroids represent average distances between treatments (or time points) across all sampling events. For details of differences between treatments, see statistical analysis and results for PERMANOVA in Table 3.

Disturbance severity had a large impact on overall community structure across the entire experimental period (Table 4, Fig. 4). While disturbance frequency did not alter community structure when compared to the single #3 treatment (Fig. 4a), frequency and severity of disturbance interacted to influence community structure in comparison to single #1 (Table 4, Fig. 4b).

**Table 4:** Results from partly-nested PERMANOVA on all algal taxa surveyed in experimental plots.

Source	df	M versus 3		M versus 1	
		MS	P(perm)	MS	P(perm)
Frequency (F)	1	738.88	0.406	1,368.3	0.073
Severity (S)	1	8,143	**0.001**	9,009.2	**0.001**
FxS	1	2,478.3	0.058	1713.7	**0.038**
Plot(FxS)	16	793.57	**0.029**	537.32	0.461
Residual	100	550.56		535.00	

The term “Plot(F × S)” tests for the effects of differences over time. The first analysis (M versus 3) compares the % cover of taxa between the multiple small disturbance treatment and the disturbance at time point 3. The second analysis (M versus 1) compares the % cover of taxa between the multiple small disturbance treatment and the disturbance at time point 1. % cover was measured initially and then at 2, 4, 6, 12, 18 months post final disturbance after disturbance. P values < 0.05 are in bold.

## Discussion

This study provides new insights into how a little tested component of disturbance ecology—different frequencies of disturbance that result in the same net-severity of disturbance—can influence population and community dynamics. *Ecklonia* recruits from the first year showed greater recruitment in the triple-disturbance treatments, but this varied with the severity of disturbance, with the highest recruitment with 50% disturbance. As a result of the increased recruitment and subsequent growth of *Ecklonia* recruits, the greatest increases in cover of *Ecklonia* were also in the triple-disturbance 50% disturbance treatment. As an ecosystem engineer, this effect on *Ecklonia* had knock-on effects through the entire benthic community.

The effect of the triple disturbance treatment on percentage cover of *Ecklonia* manifested later in the experiment (12–18 months), which persisted despite periods of convergence (12 month survey). The influence of the triple disturbance treatment on *Ecklonia* recruitment did not continue into the subsequent (year 2) recruitment season. However, given that recruitment in *Ecklonia* is usually strongly stimulated by disturbance to the canopy^50,55^, there was an initial facilitative effect of *Ecklonia* adults on new recruits in the following two months after the first disturbance that continued over the period of the experiment. This resulted in a greater number of recruits that survived into the juvenile stage and resulted in the increased percentage cover of *Ecklonia* and canopy-forming fucoids in the triple-low disturbance treatment at 18 months.

There are a number of potential mechanistic explanations for how smaller but more frequent disturbances might improve *Ecklonia*’s recruitment and recovery over the period of this experiment. One possible explanation for the facilitative effect of *Ecklonia* adults on recruits lies in the risk to recruits of being adversely affected by the dramatic change in environmental or abiotic conditions resulting from the loss of a dense canopy^47,49,56,57^. Toohey and Kendrick’s (2007)^58^ study of the survival of juvenile *Ecklonia* after disturbance observed that very few individuals suffered mortality from dislodgement (wave action), but many were observed with a bleached stipe and holdfast, indicative of light stress. While gametophytes need a certain light level to stimulate reproduction (meaning that removing a canopy can increase recruitment), young sporophytes are more susceptible to chronic photoinhibition with photodamage (500 μmol m^−2^ s^−1^ PAR for *Ecklonia cava* from^59^). Light levels at various sites in Port Phillip Bay can often reach light levels of between 400 and 500 μmol m^−2^ s^−1^ PAR over the summer period at depths similar to our study site^60,61^. If this is the case, one explanation could be that the triple-low disturbance allowed recruits to slowly acclimate to the new environmental conditions, including higher light intensity. It also stands to follow, that this effect would be more pronounced in the treatment where some *Ecklonia* were still present (50% disturbance) in comparison to a complete removal (100%).

As *Ecklonia* is the competitively dominant alga in the community, the abundance of other species is tightly linked to its abundance^40,62^. This is evident from the increase in cover of canopy-forming fucoids as canopy-space was opened up in the initial months following disturbance. However, as *Ecklonia* recovery slowed many of these effects began to disappear. Here, the disturbance regime appeared to facilitate sea urchins to maintain open space and inhibit some recovery. This effect, of the disturbance regime driving a shift from kelp bed to ‘urchin barren’ without an increase in sea urchin density has been reported previously^41,63,64^. Here, in the face of this shift, plots with triple disturbances with a net-severity of 50% were able to show signs of recovery and resilience.

The response of ecosystems to disturbance depends on characteristics of the disturbed patch, the morphological and reproductive traits of species present at the site to grow back or recruit into this space and the reproductive biology of species not present^11,65,66^. For the species present, less severe disturbances may not cause plant mortality, in which case recovery of native species can occur relatively quickly from plant growth or re-growth^67-69^. However, if mortality does occur from the disturbance, then organisms must rely on the storage effect to recover from disturbance^66^, manifesting from either the seedbank or on recruitment from neighbouring sites^70-72^. In kelp forests more broadly, the storage effect has been responsible for aiding recovery and promoting resilience up to two years post ENSO-related die back^73^. However, extreme or extended (both spatially and/or temporally) disturbance events may inhibit this ability and drive shifts in ecosystem state as observed in recent examples in Western Australia^4^ and California^74^.

Community shifts due to changes in disturbance regime may also result from species not initially present, recruiting into the site post-disturbance^11^, in some cases resulting in invasion and dominance by non-indigenous species^75-77^. This is also even more likely when physical disturbance might be combined with another stressor or different components of the disturbance regime^78,79^. In an *Ecklonia* dominated kelp ecosystem, a combination of physical disturbance to the native kelp and increased nutrients acted synergistically to result in dominance of the introduced Japanese kelp *Undaria pinnatifida*^50^. Similarly, in an arid grass ecosystem, different disturbance pathways (from either increasing fire frequency or high severity fires) can lead to dominance by invasive grasses^80^. In this way, altered disturbance regimes are likely to promote change in native and non-indigenous species composition^76^.

The results presented in this study extend the results of other studies that show more severe or novel disturbance regimes can cause a shift in community dynamics and decline in ecosystem condition^80,81^. This can occur with changes in the frequency or severity of fires^80^, storm events^81,82^ or anthropogenic disturbances^21^. Importantly, that it is not just the net severity of disturbance, but the synergistic effect of the frequency of disturbances and the severity of each disturbance^80^. With both frequency or severity of disturbances predicated to increase (and in some cases both) due to climate change, understanding and predicting the nature of their interaction will be critical^83-86^. This study and others indicate we may indeed see an erosion of the resilience of ecosystems and shifts from one community state to another as a result of unanticipated effects of disturbances that change in several directions^81^.

This study reveals the importance of considering the combination of frequency and severity of disturbance when we measure the impact of disturbance regimes. While we recognise how individual components of disturbance influence species and community dynamics^29,33,51^, the results of our study mean that the design of disturbance experiments is critical to understanding responses to disturbance in natural communities. For example, many disturbance treatments are applied at one point in time, which may represent a novel and unusual disturbance regime. Failure to consider both magnitude and frequency of disturbance in the context of the “natural” disturbance regime may lead to misinterpretation of community responses to disturbance.

While much prior research has examined disturbance impacts under the assumption that “disturbance” is a single factor, evidence^18,87,88^ to support disturbance theory^11,65^ has grown that disturbance is a multi-factorial process. For example, a range of studies reveal high levels of ‘noise’ or unpredictability, raising questions about the usefulness of our current theoretical principles^19^. Here, we show how two different components of disturbance (frequency and severity) can interact to influence community dynamics and resilience. Fortunately, there are increasing numbers of theoretical advances that tie together the different components of disturbance in a more holistic framework^8,82^. From a number of recent studies, it has become clearer than ever that we need to consider multiple facets of disturbance and the properties of that community to predict or understand the true influence of a disturbance regime on ecosystem dynamics^8,89^.

## Supplementary information

Supplementary file 1 (DOCX 69 kb)

## Data Availability

All data uploaded to CloudStor.
