# Supplementary material for: More severe disturbance regimes drive the shift of a kelp forest to a sea urchin barren in south-eastern Australia
Source: Sci Rep. 2020 Jul 9;10:11272. doi: 10.1038/s41598-020-67962-y (PMC7347924; doi:10.1038/s41598-020-67962-y)
Supplement: Supplementary file 1 — (DOCX 69 kb) [file 41598_2020_67962_MOESM1_ESM.docx]

# **More severe disturbance regimes drive the shift of a kelp forest to a sea urchin barren in south-eastern Australia**.

# Appendix

**Carnell, Paul .E^1,2^* and Keough, Michael .J^1^**

^1^ School of BioSciences, The University of Melbourne, Parkville, Victoria, 3010.

^2^ School of Life and Environmental Science, Centre for Integrative Ecology, Deakin University, Burwood, Victoria 3125

[*paul.carnell@deakin.edu.au](mailto:*paul.carnell@deakin.edu.au), [mjkeough@unimelb.edu.au](mailto:mjkeough@unimelb.edu.au)

Table A1. Results of Maculy’s test of Sphericity on % cover of *Ecklonia radiata* over the duration of the 18 month experiment.

| Within Subjects Effect | Mauchly's W | Approx. Chi-Square | df | Sig. | Epsilon^c^ |
| --- | --- | --- | --- | --- | --- |
|  |  |  |  |  | Greenhouse-Geisser |
| Time | .109 | 66.620 | 14 | <0.001 | .575 |

Table A2. Results of Levene’s test of equality of error variances on % cover of *Ecklonia radiata* at each time point over the duration of the 18 month experiment. Df = 7 and 32.

|  | | Levene Statistic | Sig. |
| --- | --- | --- | --- |
| Logit 0 months | Mean | 5.856 | .000 |
|  | Median | 2.067 | .077 |
| Logit 2 months | Mean | 4.268 | .002 |
|  | Median | 2.053 | .078 |
| Logit 4 months | Mean | 5.020 | .001 |
|  | Median | 3.714 | .005 |
| Logit 6 months | Mean | 2.314 | .050 |
|  | Median | 1.260 | .301 |
| Logit 12 months | Mean | 4.840 | .001 |
|  | Median | 1.342 | .263 |
| Logit 18 months | Mean | 6.653 | .000 |
|  | Median | 2.196 | .061 |

Figure A1. Average percentage cover over time (months post-disturbance) of a) *Ecklonia radiata* b) canopy-forming fucoids c) Turf-forming Ectocarpales d) Sessile invertebrates and the interaction between severity (50% or 100%) and frequency (triple-low & single #1) or between timing controls (single #1,#2,#3) of disturbance. Solid lines and filled symbols = 50% removals; dotted lines and unfilled symbols = 100% removals. Squares = #1, Circles = #2, Diamonds = #3 and Triangles = triple disturbances. Black lines = single disturbances at time point #3 or #1, while Grey lines = triple disturbances or single disturbance at time point #2.

Figure A2. Average percentage cover over time (months post-disturbance) of a) Filamentous red algae b) Encrusting coralline algae (Encrusting), c) Dictyotales and d) Sediment and the interaction between severity (50% or 100%) and frequency (triple-low & single #1) or between timing controls (single #1,#2,#3) of disturbance. Solid lines and filled symbols = 50% removals; dotted lines and unfilled symbols = 100% removals. Squares = #1, Circles = #2, Diamonds = #3 and Triangles = triple disturbances. Black lines = single disturbances at time point #3 or #1, while Grey lines = triple disturbances or single disturbance at time point #2.

Figure A1.

**Triple Vs Single #1**

**Timing Controls: Single #1,#2,#3**

Figure A2.

**Triple Vs Single #1**

**a**

**b**

**c**

**d**

**Timing Controls: Single #1,#2,#3**
